# E-cigarettes in young people: applying the precautionary principle in primary care

**DOI:** 10.3399/bjgp23X734997

**Published:** 2023-09-28

**Authors:** Ichechim White, Liz Hare, Marian Davis, Stephanie Lamb, Emma Park, Lucy White, Faraz Mughal

**Affiliations:** Villa Street Medical Centre; Member, Royal College of General Practitioners (RCGP) Adolescent Health Group, RCGP, London; Weston-super-Mare; RCGP Adolescent Health Group, RCGP, London; Herefordshire; RCGP Adolescent Health Group, RCGP, London; International Association for Adolescent Health; Herne Hill Group and the Well Centre; RCGP Adolescent Health Group, RCGP, London; Primary Care Sheffield, RCGP Adolescent Health Group, Sheffield; Medway Medical School, University of Kent, Canterbury; School of Medicine, Keele University, Keele; RCGP Adolescent Health Group, RCGP, London

## National Priority

In 2019 the UK Government set out to make England smokefree (smoking prevalence of 5% or less) by 2030.^1^ The Tobacco Control Plan for England states smoking as the single largest cause of preventable deaths, with over 7 million people still smoking in England.^2^

This plan also sets out to *‘eliminate smoking among under 18s and achieve the first smokefree generation’*.^2^ An independent ‘Khan review’ into the smokefree policy in 2022 set out 15 recommendations to support smoking cessation.^3^ Crucially, promoting the use of e-cigarettes in existing smokers has been suggested as a key element to achieve the smokefree target.^3^ The free ‘swap to stop’ packs launched in April 2023 aims to provide 1 million cigarette smokers with e-cigarettes in exchange for cigarettes. The Khan review did acknowledge the need to reduce young people’s uptake of e-cigarettes by banning child-friendly labelling and packaging. In the UK, selling e-cigarettes to under 18 year olds is illegal.^4^

Despite recognition that e-cigarettes should not be targeted at adolescents, there are growing health and environmental concerns over e-cigarette use in young people. This editorial highlights the concerns about adolescents, and offers support to primary care practitioners when assessing e-cigarette use in young people.

## What are E-Cigarettes?

E-cigarettes are electronic nicotine delivery systems or electronic non-nicotine delivery systems. They require heating, vaporising an e-liquid mixture via lithium battery power, so it can be inhaled. The e-liquid consists most commonly of water, propylene glycol, nicotine, and flavourings. There are over 7000 flavours.^5^

## Young People and E-Cigarettes

E-cigarette use, commonly known as vaping, among the adolescent population appears to be increasing. In 2023, around 21% of 11–18 year olds in Great Britain had tried vaping, an increase from 13.9% in 2020 before the first lockdown. Around 8% of young people are currently using e-cigarettes.^6^ There are concerns that young people can perceive e-cigarettes as harmless: the main reason of use in those who had never smoked was ‘to give it a try’ while recognising a lack of awareness of ingredients and their effects.^4^

## A New Ecological Threat

Disposable e-cigarette models (which are pre-filled with liquid and used only once) are the most popular type of vaping device and their use is increasing. In 2022, just over half of 11–18 year olds in Great Britain who vaped used disposables, while 18.7% used tank models (reusable and rechargeable kits).^4^

The *Lancet* recently highlighted environmental concerns with regards to incorrect vape disposal due to the release of plastic, electronic, and hazardous chemical waste into the environment.^7^ Users can be potentially unaware of the need for recycling, and disposable vapes are designed in such a way that they can be difficult to disassemble.^7^ The Royal College of Paediatrics and Child Health has called for a ban of all disposable e-cigarettes.^8^

It is widely accepted that the marketing of e-cigarettes appears to target adolescents with newer, cheaper disposable products, a multitude of flavours available, and placement close to confectionary by vendors. In Australia, the sale of non-prescribed, nicotine-containing e-cigarettes is to be banned in young people, which includes disposable vapes.^9^

## Nicotine and the ‘Gateway Effect’

There is debate as to whether use of e-cigarettes increases initiation of tobacco smoking or use of other drugs, termed as the ‘gateway effect’.^10^ There are concerns that early exposure to nicotine through e-cigarette use could drive addictive patterns in the susceptible adolescent brain. An ongoing Cochrane review aims to assess the relationship between e-cigarette use and later cigarette smoking in young people and should yield important findings.^10^

The US outbreak of e-cigarette vaping associated lung injury (EVALI) from 2019–2020 reflects the need for clinicians to remain vigilant to potential complications from use and report these to the Medicines and Healthcare products Regulatory Agency via the yellow card system.^11^ In these cases, vitamin E acetate and tetrahydrocannabinol (THC) were additives implicated, with a small proportion of cases (11%) where nicotine liquids were only used.^11^

## Communication with Young People

Primary care clinicians can discuss e-cigarette use with young people, supporting and signposting to smoking cessation services. We provide areas for clinicians to consider when consulting young people (aged 11–25 years):

1. Attempt to assess young people alone (explain confidentiality and avoid judgemental statements).
2. Consider routinely asking AND recording e-cigarette use as part of a holistic health screen, for example, discussing use of any external substances, as part of ‘Drugs’ in the HEEADSSS framework (Home, Education/Employment, Eating, Activities, Drugs, Sexuality, Suicidal ideation and Safety).^12^
3. Enquire, where appropriate, if the young person is an existing cigarette smoker or e-cigarette user: Why and how long? What do they vape, for example, strength and product? Consider illicit substance misuse, such as THC, spice, or black market e-liquids.
4. Check where/who the young person obtains e-cigarettes from. If under 18 years, consider if there is a safeguarding concern and discuss with the young person if there is a need to share due to risk, vulnerability, or age.
5. Check knowledge about e-cigarettes (components and potential harms, such as EVALI).
6. Highlight the ecological harm from disposable vape use. This may motivate young people to stop on grounds of climate change. For those over 18 years using for cessation, suggest rechargeable devices.
7. Consider as an alternative to tobacco smoking in those over 18 years struggling with cessation. Remember ‘swap to stop’ schemes and signposting to local support services.

## A Public Health Challenge

The UK Government has just announced steps to reduce marketing of e-cigarettes to those under 18 years. National guidance and policy supports the use of nicotine-containing e-cigarettes as a stop-smoking intervention.^3,13^ In a recent international systematic review, GPs described mixed views on supporting the use of e-cigarettes as a smoking cessation aid, with some GPs hesitant to do so.^14^ Although suggested as a key component of smoking cessation, primary care clinicians appear to remain unsure, and this identifies the need for ongoing evidence-informed education and training.

## The Precautionary Principle

E-cigarette use in young people is rising and this is worrying given the possible risk of addiction and unknown long-term effects. The evidence base at present is inconclusive about the long-term impacts of e-cigarettes and there are concerns over possible harmful ingredients. National guidance suggests nicotine-containing e-cigarettes should be a stop smoking intervention option but only in adults (over 18 years).

Primary care clinicians are thus in a difficult position: needing to balance the offer of cessation to young adult smokers where appropriate with vigilant prevention of e-cigarette uptake in non-smoking young people. Clinicians should communicate the possible benefits and risks of e-cigarette use with young smokers in a personalised approach to smoking cessation. In non-smokers and primary users of e-cigarettes, clinicians are encouraged to use a proactive health promotion approach to attempt to reduce use.
